# Iron replacement therapy in heart failure: a literature review

**DOI:** 10.1186/s43044-021-00211-3

**Published:** 2021-09-26

**Authors:** Hassan Ismahel, Nadeen Ismahel

**Affiliations:** University of Glasgow, University Avenue, Glasgow, G12 8QQ UK

**Keywords:** Iron deficiency, IV iron, Iron replacement, Ferrous carboxymaltose, Iron sucrose, Heart failure, Heart failure with reduced ejection fraction

## Abstract

**Background:**

Heart failure (HF) is a major global challenge, emphasised by its designation as the leading cause of hospitalisation in those aged 65 and above. Approximately half of all patients with HF have concurrent iron deficiency (ID) regardless of anaemia status. In HF, iron deficiency is independently associated with higher rates of hospitalisation and death, lower exercise capacity, and poorer quality-of-life than in patients without iron deficiency. With such consequences, several studies have investigated whether correcting ID can improve HF outcomes.

Main body.

As of 1st June 2021, seven randomised controlled trials have explored the use of intravenous (IV) iron in patients with HF and ID, along with various meta-analyses including an individual patient data meta-analysis, all of which are discussed in this review. IV iron was well tolerated, with a comparable frequency of adverse events to placebo. In the context of heart failure with reduced ejection fraction (HFrEF), IV iron reduces the risk of hospitalisation for HF, and improves New York Heart Association (NYHA) functional class, quality-of-life, and exercise capacity (as measured by 6-min walk test (6MWT)) distance and peak oxygen consumption. However, the effect of IV iron on mortality is uncertain. Finally, the evidence for IV iron in patients with acute decompensated heart failure, or heart failure with preserved ejection fraction (HFpEF) is limited.

**Conclusions:**

IV iron improves some outcomes in patients with HFrEF and ID. Patients with HFrEF should be screened for ID, defined as ferritin < 100 µg/L, or ferritin 100–299 µg/L if transferrin saturation < 20%. If ID is found, IV iron should be considered, although causes of ID other than HF must not be overlooked.

## Background

Unquestionably, heart failure (HF) is a major global challenge with an estimated prevalence of 3–20 cases/1000 population [1]. This increases substantially to more than 100 cases/1000 population in those aged 65 and over, and is the leading cause of hospitalisation in this age group [1, 2]. This carries significant health and economic ramifications despite numerous advances in HF management, with patients limited by worsening symptoms, exercise intolerance, and an increased risk of recurrent hospitalisation and mortality [3–5]. Moreover, approximately 50% of patients with HF have concurrent iron deficiency (ID) with or without anaemia [3, 6–8]. With such high prevalence, further studies are being conducted to determine whether targeting ID can improve HF outcomes. This review summarises normal iron homeostasis, the pathophysiology and consequences of ID in HF, and current evidence surrounding iron replacement, before discussing guideline recommendations for iron replacement therapy in patients with HF.

## Main text

### Iron functions and homeostasis

Iron plays a critical role within various cell systems thanks to its ability to readily undergo redox cycling between its two primary oxidative states, ferric (Fe^3+^) and ferrous (Fe^2+^) iron [9–11]. Indeed, iron is an important cofactor for various enzymes, acting as a catalyst for important biochemical reactions during oxygen uptake, transport, storage and oxidative metabolism [11, 12]. Most notably, iron is an obligate component of haemoglobin (Hb) allowing it to transport oxygen around the body [13]. Additionally, iron is a key component of mitochondria, necessary for energy production [6–12]. Given the significant role of iron within every mammalian cell, it becomes apparent why treating ID might be beneficial, especially given its high prevalence amongst heart failure patients.

**Fig. 1:**
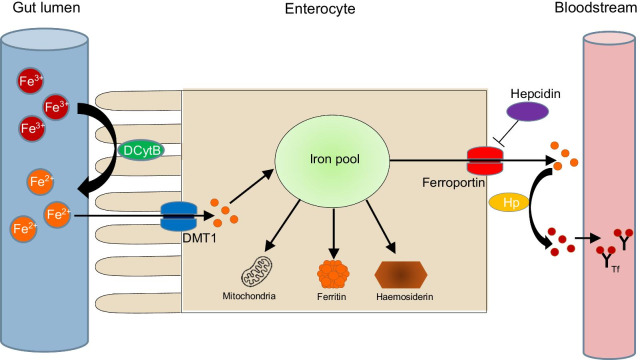
Molecular pathway of iron absorption and homeostasis. Fe^3+^  = ferrous iron; Fe^2+^  = ferric iron; DCytB = duodenal cytochrome B; DMT1 = divalent metal transporter 1; Hp = hephestin; Tf = transferrin

Due to the absence of a physiological mechanism to excrete excess iron in mammals, iron homeostasis is regulated by a combination of iron absorption in the duodenum and proximal jejunum, illustrated in Fig. 1; accompanied by iron release from macrophages of the reticuloendothelial system (RES) via a delicate interaction between the hormone hepcidin and the transmembrane protein ferroportin [8, 11, 12]. Iron can either exist intracellularly in its ferrous form (Fe^2+^) where it is stored as ferritin or haemosiderin; or extracellularly in its ferric form (Fe^3+^) where it circulates bound to transferrin. Ferroportin is the key transmembrane protein controlling the distribution of iron by exporting the iron stored within enterocytes and RES macrophages into circulating plasma, increasing transferrin saturation (TSAT). Conversely, hepcidin is a negative regulator of iron release by binding to ferroportin and degrading it, reducing dietary iron absorption by enterocytes and iron release from RES macrophages, ultimately reducing iron levels. The interaction between hepcidin and ferroportin is the single most important regulator of total body iron [8–11].

It must be noted that total body iron exists within a narrow therapeutic window, and that iron deficiency, or iron overload (such as in haemochromatosis) can be detrimental. Although the average western diet contains 15-20 mg/day, a single human requires only 1-2 mg of iron daily to compensate for the daily loss via uncontrollable mechanisms, most notably enterocyte death in both sexes, and blood loss secondary to menstruation in females. Normally, total body iron stores are approximately 4 g, with 75% (3 g) utilised by erythroblasts to synthesise haemoglobin, emphasising iron’s critical role in oxygen transportation [8, 14, 15].

### Definitions of iron deficiency

Iron deficiency is recognised as the most common nutritional deficiency worldwide, affecting one-third of the global population [6, 8]. Iron deficiency can be classified as absolute or functional. Absolute iron deficiency is defined as serum ferritin < 100 μg/L and refers to decreased total body stores mainly secondary to gastrointestinal blood loss, impaired gastrointestinal absorption, or poor appetite or nutrition [16, 17]. Conversely, functional iron deficiency is defined as serum ferritin 100-299 μg/L, with transferrin saturation < 20%. Unlike absolute ID, functional ID refers to normal or elevated total body iron stores which are theoretically capable of meeting body demand, but are unavailable for incorporation into erythroid precursors since they cannot be exported from the intracellular compartment [6, 16, 17]. The latter is typically seen in anaemia of chronic disease due to elevated hepcidin levels, inhibiting the ability of ferroportin to export intracellular iron stores, thereby resulting in reduced iron utilisation in important cellular processes including erythropoiesis [16–18].

Risk factors for ID in heart failure include elevated levels of N-terminal pro–B-type natriuretic peptide (NT-proBNP) and C-reactive protein, female sex, and advanced disease [8]. It is important to emphasise that many HF patients develop ID without being anaemic, hence screening for ID is crucial, even in the presence of normal Hb values [11]. This is commonly via measurement of serum ferritin and TSAT. However, serum ferritin is an acute-phase reactant that can be elevated during periods of inflammation, and values must be interpreted in the context of the patient’s clinical condition. Bone marrow aspiration with Prussian blue staining is gold standard for defining ID, but is understandably invasive and expensive. The current ID definition relies on ferritin levels < 100 µg/L, or 100–300 µg/L with TSAT < 20% and carries a sensitivity of 82% and specificity of 72% for true ID. However, one study noted that a definition based on a single parameter of TSAT ≤ 19.8% or serum iron ≤ 13 μmol/L better correlated with absolute or functional ID (sensitivity 94% for both, specificity 84% and 88% respectively; *p* < 0.05). Additionally, TSAT ≤ 19.8% and serum iron ≤ 13 µmol/L (and not ferritin) were independent predictors of mortality in HF patients, providing prognostic values [19, 20].

### Pathophysiology and consequences of iron deficiency in heart failure

Regardless of anaemia, iron deficiency plays a key role in heart failure since cardiac myocytes have high metabolic activity, making them particularly vulnerable to decreased iron levels. A small study noted a reduction in intracellular iron stores within cardiomuocytes of explanted failing hearts compared to normal myocardium (0.49 ± 0.07 μg/g vs. 0.58 ± 0.09 μg/g, *p* < 0.05). This was supported by a 68% reduction in transferrin receptor 1 (Tfr1) mRNA expression in the cardiomyocytes of the failing heart compared to the nonfailing heart (*p* < 0.05). Given the crucial role of the transferrin receptor as the major uptake pathway for iron into myocardium, its downregulation in heart failure partly explains why cardiac iron stores were reduced. This likely occurs in response to increased levels of catecholamines and aldosterone, both of which are commonly seen in HF [6, 21].

The prevalence of intracellular ID in human cardiomyocytes in patients with advanced heart failure was further studied in left ventricular samples obtained from 91 consecutive HF patients undergoing transplantation and 38 HF-free organ donors (used as healthy controls) [22]. Myocardial iron content was lower in HF compared with controls (156 ± 41 vs. 200 ± 38 µg·g-1 dry weight, P < 0.001), and ID was independent of anaemia. Cardiomyocyte ID was associated with reduced mitochondrial function, especially diminished citric acid cycle enzyme activity, and decreased expression of enzymes protecting against oxidative stress. The potential consequences of intracellular ID was also studied in human embryonic stem cell-derived cardiomyocytes by *Hoes *et al*.* These investigators demonstrated that chemically induced ID directly impaired mitochondrial respiration, with a reduction in cellulatr ATP levels by 74% (*p* < 0.0001). ID also reduced contractile force and the maximum velocities during both systole and diastole. These effects were reversible following restoration of intracellular iron levels [23].

The pathophysiology underlying ID in HF is multifactorial, with hepcidin recognised as an important contributor. Normally, during times of absolute ID, hepcidin is down-regulated to increase the ferroportin-mediated processes of iron absorption by enterocytes and iron release by RES macrophages [11, 17]. However, chronic inflammatory states such as heart failure trigger prolonged release of inflammatory mediators that depress bone marrow function whilst concurrently stimulating hepatocytes of the liver to synthesise more hepcidin. It is this upregulation of hepcidin that inhibits the release of iron from body stores, resulting in a pattern of functional, not absolute, ID despite seemingly adequate total body iron stores. The upregulation of hepcidin partly explains why oral iron supplements are ineffective in patients with ID and HF [6, 8–11, 17].

Iron deficiency in the context of heart failure is widely recognised as an independent predictor of poorer outcomes including fatigue, reduced exercise capacity, reduced quality of life (QoL), increased hospitalisation, and increased mortality [3, 6–8, 24–27]. In a prospective observational study conducted by *Jankowska *et al. in 546 HF patients, ID was a strong and independent predictor of death and heart transplant, regardless of anaemia [28]. This finding was reinforced by a study of 157 HF patients by *Okonko *et al. in which ID was associated with a three-fold higher risk of mortality, irrespective of anaemia status [27]. Additionally, ID has been shown to be an independent predictor of exercise capacity in HF patients, with lower peak oxygen consumption (VO_2_ max) compared to those without ID [11, 29]. Similarly, HF patients with ID underperform in submaximal exercise tests such as the 6-min walk test (6MWT) when compared to those without ID [16, 30]. Various studies have also reported that ID is independently associated with QoL, for example predicting a higher score with the Minnesota Living with Heart Failure Questionnaire [11, 31, 32]. Notably, it has also been demonstrated that a greater prevalence of ID appears to correlate with higher (i.e., worse) New York Heart Association functional class [7, 27, 33].

### Intravenous iron therapy in heart failure

#### IV iron in heart failure with reduced ejection fraction: evidence from randomised controlled trials

Given the significance of iron deficiency in heart failure, numerous studies have investigated the possibilities for correcting it. As of 1^st^ June 2021, seven RCTs comparing IV iron with placebo in patients with HF were completed, designated *Toblli *et al. [34]*,* FERRIC-HF [24], FAIR-HF [35], CONFIRM-HF [36], EFFECT-HF [37], PRACTICE-ASIA-HF [38] and AFFIRM-AHF [39]. A comparison of each trial is shown in Table 1. A total of 2,164 patients were enrolled, with 1166 (53.9%) randomly allocated IV iron and 998 (46.1%) allocated to placebo. Ferric carboxymaltose (FCM) was used in five trials [35–39] and iron sucrose in the other two (IS) [24, 34].

**Table 1:** Current published randomised controlled trials investigating intravenous iron in heart failure

	Toblli et al	FERRIC-HF	FAIR-HF	CONFIRM-HF	EFFECT-HF	PRACTICE-ASIA-HF	AFFIRM-AHF
Publication year	2007	2008	2009	2014	2017	2018	2020
HF diagnosis	HFrEF	HFrEF	HFrEF	HFrEF	HFrEF	Acute decompensated HF	Acute decompensated HF
Number of participants	40	35	459	301	172	49	1108
Randomisation and preparation	1:1 Iron sucrose: placebo	2:1 Iron sucrose: placebo	2:1 FCM:placebo	1:1 FCM:placebo	1:1 FCM:placebo	1:1 FCM:placebo	1:1 FCM:placebo
Blinding	Double-blind	Double-blind	Double-blind	Double-blind	Open-label	Single-blind	Double-blind
Primary outcomes	Change in NT-proBNP level and CRP	Change in absolute pVO_2_ (ml/kg/min) from baseline to week 18	Changes in self-reported Patient Global Assessment and NYHA class from baseline to week 24	Change in 6-MWT distance from baseline to week 24	Change in peak VO_2_ (ml/min/kg) from baseline to week 24	Change in 6-MWT distance over 12 weeks	Composite of total HF hospitalisations and CV death up to 52 weeks after randomisation
Duration of follow-up	24 weeks	18 weeks	24 weeks	52 weeks	24 weeks	12 weeks	52 weeks
Dosage	IV Iron sucrose 200 mg weekly for 5 weeks	IV Iron sucrose 200 mg weekly until ferritin > 500 ng/ml, then 200 mg monthly thereafter for a total of 16 weeks	IV FCM 200 mg weekly until iron repletion achieved, then every 4 weeks thereafter	IV FCM 500–2000 mg based on Hb and weight	IV FCM 500–1000 mg based on Hb and weight	Single dose of 1000 mg IV FCM	500–1500 mg FCM based on Hb and weight
Important inclusion criteria	LVEF ≤ 35% NYHA II–IV Hb < 12.5 g/dL for men and < 11.5 g/dL for women	LVEF ≤ 45% NYHA II–III pVo_2_/kg ≤ 18 ml/kg/min Hb < 12.5 g/dL anaemic group; 12.5–14.5 g/dL non-anaemic group	LVEF ≤ 45% NYHA II–III Hb between 9.5 and 13.5 g/dL	LVEF ≤ 45% NYHA II–III Elevated natriuretic peptides Hb < 15 g/dL	LVEF ≤ 45% NYHA II–III Elevated natriuretic peptides Peak VO_2_ of 10–20 mL/kg/min	Hospitalised for HF Hb ≤ 14 g/dL	LVEF < 50% Hospitalised for HF
Definition of ID	Serum ferritin < 100 ng/ml and/or with transferrin saturation (TSAT) ≤ 20%	Serum ferritin < 100 μg/l, or between 100 and 300 μg/l if TSAT < 20%	Serum ferritin < 100 μg/L, or between 100 and 299 μg/L if the TSAT < 20%	Serum ferritin < 100 ng/mL, or between 100 and 300 ng/mL if TSAT < 20%	Serum ferritin < 100 ng/mL. or between 100 and 300 ng/mL if TSAT < 20%	Serum ferritin < 300 ng/mL if transferrin saturation is < 20%	Serum ferritin < 100 ng/mL, or 100–299 ng/mL if TSAT < 20%

*HF* heart failure, *CV* cardiovascular, *HFrEF* heart failure with reduced ejection fraction, *FCM* ferrous carboxymaltose, *6-MWT* 6-min walk test, *TSAT* transferrin saturation, *NYHA* New York Heart Association, *LVEF* left ventricular ejection fraction, *NT=proBNP* N-terminal prohormone of brain natriuretic peptide, *Hb* haemoglobin, *IV* intravenous

The first RCT exploring the use of IV iron in HF patients was conducted in 2007 by *Toblli *et al. in 40 HF patients with iron deficiency anaemia (defined as a Hb of < 12.5 g/dL in men and < 11.5 g/dL in women), in addition to a LVEF of ≤ 35% and serum ferritin of < 100 ng/ml and/or with transferrin saturation (TSAT) ≤ 20%. Half were allocated to IV iron sucrose and half allocated saline, with those given iron demonstrating a reduction in NT-proBNP level, improvements in LVEF, improvements in 6MWT distance and fewer hospitalisations throughout a 6 month follow-up period (0 in iron group vs 5 in control) [34]. *Okonko *et al. expanded upon this in 2008 with FERRIC-HF, a randomised study involving 35 patients with concurrent HF and ID, including those with and without anaemia, demonstrating improvements in exercise capacity, patient global assessment (PGA) scores, and NYHA functional class. IV iron sucrose was well tolerated, with similar adverse events (AE) profiles between both groups [24].

The first relatively large-scale, purpotedly double-blind multicentre trial was published in 2009 by *Anker *et al*.* In FAIR-HF (Ferinject Assessment in patients with IRon deficiency and chronic Heart Failure trial), 459 HF participants in NYHA functional class II or III, with LVEF ≤ 40–45%, ID (defined as ferritin of either < 100 μg/L, or between 100 and 299 μg/L if TSAT < 20%) and a Hb concentration of 95–135 g/dL, were randomised in a 2:1 ratio to either IV FCM or saline and followed up for 24 weeks. True blinding was not possible because the IV iron and saline solutions differed in colour, however measures like black syringes and curtains were used to shield the solutions. Of those given IV FCM, 50% reported an improvement in the primary outcome of patient global assessment, compared to 28% of patients allocated placebo (odds ratio [OR] for improvement: 2.51; 95% confidence interval [CI]: 1.75–3.61; *p* < 0.001). There were also statistically significant improvements in secondary outcomes, including in NYHA functional class, 6MWT distance, and QoL quantified using the Kansas City Cardiomyopathy Questionnaire. These benefits were observed regardless of anaemia status at baseline. However, there were no significant changes to all-cause mortality (3.4% in FCM vs. 5.5%, in placebo) or first hospitalisations (17.7% in FCM vs 24.8% in placebo). There was no significant difference between treatment arms with respect to AEs, suggesting IV FCM was safe for use with no unacceptable side effects [35].

A second trail, CONFIRM-HF (Ferric CarboxymaltOse evaluatioN on perFormance in patients with IRon deficiency in coMbination with chronic Heart Failure) was published in 2015. This was also described as a double-blind, multicentre, prospective RCT and had similar objectives to FAIR-HF and obtained similarly encouraging results, although also suffered from incomplete blinding. A total of 301 ambulatory patients with a LVEF of ≤ 45%, NYHA functional class II or III, an elevated NT-proBNP or B-type natriuretic peptide (BNP) and ID were enrolled. Patients were randomised 1:1 to either IV FCM or placebo and followed for 52 weeks. The primary endpoint was the change in 6MWT distance from baseline to week 24. Patients receiving IV iron achieved a greater 6MWT distance than those receiving placebo (33 m ± 11 m more; *p* = 0.002) at week 24. This benefit persisted at week 52 (36 ± 11 m; *p* < 0.001). Additionally, NYHA class, PGA scores, QoL scores and fatigue scores improved, significantly, from week 24 onwards. Moreover, the risk of hospitalisation for HF was lower in the FCM, compared to placebo, group (hazard ratio [HR] 0.39, 95% CI 0.19–0.82; *p* = 0.009) although this finding was based on small numbers [36]. This trial reaffirmed the findings obtained from FAIR-HF, and raised the possibility that iron therapy might reduce HF hospitalisation.

More recently, the Effect of Ferric Carboxymaltose on Exercise Capacity in Patients with Chronic Heart Failure and Iron Deficiency (EFFECT-HF) study reinforced the positive results obtained from earlier trials. This study randomised 174 patients with HF and ID to either IV FCM or standard care, without blinding of treatment assignment. The results, published in 2017, suggested patients receiving IV FCM maintained their baseline peak oxygen consumption (peak VO_2_) after 24 weeks (least-square mean change: − 0.16 ± 0.387 ml/min/kg; *p* = 0.02) whereas those receiving standard care saw a decline in VO_2_ over this period (least-square mean change: − 1.19 ± 0.389 ml/min/kg). The between treatment difference was significant, providing more evidence that IV iron has a favourable effect on functional capacity, as compared to standard care [37].

#### IV iron in acute decompensated heart failure: evidence from randomised controlled trials

While the trials described above have shown benefit for the use of IV iron in patients with “ambulatory” (outpatient) chronic HFrEF, the effects of IV iron in patients with acute decompensated HF was unknown until recently. The AFFIRM-AHF trial, published in November 2020, has filled this gap in evidence. AFFIRM-AHF was purportedly a double-blind trial, but again did not have a placebo solution matching IV FCM. A total of 1108 patients with ID who were hospitalised with HF and had a LVEF < 50% were randomised 1:1 to either IV FCM or placebo with repeat dosing as needed for up to 24 weeks. Patients were followed for 52 weeks and the primary outcome was the composite of total hospitalisation for HF and cardiovascular (CV) death. This study narrowly missed its primary objective, with 293 primary events (57·2 per 100 patient-years) occurring in the treatment arm and 372 (72.5 per 100 patient-years) occurring in the control arm (rate ratio [RR] 0.79, 95% CI 0.62–1.01, *p* = 0·059). Moreover, there was no difference in CV death between groups (77 [14%] of 558 in FCM group vs 78 [14%] in the placebo group; HR 0·96, 95% CI 0·70–1·32, *p* = 0·81). The authors concluded that IV FCM was safe to administer in acute HF and reduced the risk of hospitalisation for heart failure, with no apparent effect on the risk of CV death [39].

The PRACTICE-ASIA-HF study recruited 50 Southeast Asian patients hospitalised for acute decompensated HF with ID, regardless of LVEF, and randomised them on a 1:1 basis to either a single dose of IV FCM 1000 mg or a single dose of IV saline before discharge. In this very small trial, a single dose of IV FCM did improve functional capacity compared to placebo. Additionally, there was no overall difference in QoL scores between either group [38].

### Evidence from meta-analyses regarding iron therapy in heart failure

A recently published meta-analysis in 2021 included all seven of the above RCTs. The aim was to evaluate whether IV iron affected the composite of hospitalisation for HF or CV mortality, as first events, in a total population of 2,166 patients (1168 assigned to IV iron; 998 assigned to control). IV iron reduced the composite of hospitalisation for heart failure or CV mortality significantly (OR 0.73; [95% CI 0.59–0.90]; *p* = 0.003) with this benefit driven by reduction in HF hospitaliastion [OR 0.67; (0.54–0.85); *p* = 0.0007], without a significant effect on CV mortality [26].

Several other meta-analyses [40–43], including an individual patient data meta-analysis involving 504 patients and 335 controls [3] have suggested IV iron confers a significant reduction in all-cause mortality, CV hospitalisation, CV mortality, and HF hospitalisation in ambulatory patients with HFrEF and ID. IV iron was also associated with significant improvements in QoL as measured by various questionnaires, NYHA functional class and 6MWT distance.

### Oral iron therapy in heart failure

Oral iron therapy, most commonly in the form of ferrous sulphate or ferrous fumarate, appear a low-cost, convenient alternative to IV iron. However, there is currently no evidence supporting their use in HFrEF, with *Gregory *et al*.* conducting a large trial titled IRONOUT HF exploring the impact of high-dose oral iron on exercise capacity (defined as a change in peak oxygen consumption, VO_2_) over 16 weeks in a randomised study with 225 participants. The study noted that high-dose oral iron had no effect on exercise capacity at 16 weeks compared to placebo (the primary objective), nor was there a significant difference in 6MWT distance, NT-proBNP levels, or Kansas City Cardiomyopathy Questionnaire between treatment arms. The authors concluded that “these results do not support use of oral iron supplementation in patients with HFrEF” [6].

Additionally, oral iron preparations have a high incidence of gastrointestinal side effects which may lead to poor compliance. Other disadvantages include poor absorption in the gastrointestinal tract secondary to intestinal oedema, poor diet, or coeliac disease for example. Moreover, upregulation of hepcidin, as seen in HF, ultimately reduces dietary iron absorption through mechanisms discussed earlier. As a result, only IV iron therapy is currently recommended [44, 45].

### Current guideline recommendations

With such promising evidence favouring the use of IV FCM in HFrEF, several guideline groups have recommended the consideration of IV iron in patients with concomitant HFrEF and ID, as seen in Table 2. In 2016, the European Society of Cardiology (ESC) released updated guidelines with a class IIa (level of evidence A) recommendation to consider IV ferrous carboxymaltose in iron-deficient patients with symptomatic HFrEF (defined as ferritin < 100 $\upmu$g/L, or ferritin 100–299 $\upmu$g/L if the TSAT is < 20%) provided other, potential causes of ID such as gastric ulcers or colon cancer, have also been investigated and treated when possible [44, 45]. Similar recommendations were made by the Scottish Intercollegiate Guidelines Network (SIGN) in 2016 [46] and the joint guidelines of the American College of Cardiology (ACC) and American Heart Association (AHA) in 2017 [47]. As of 2018, the National Institute for Health and Care Excellence has advised no recommendation for the use of iron in HF [48].

**Table 2:** Current guideline recommendations reagrding the use of intravenous iron in heart failure

Guideline group	Year of publication	Recommendation	Class/level of evidence
European Society of Cardiology [44]	2016	IV ferrous carboxymaltose should be considered in symptomatic patients with HFrEF and ID (ferritin < 100 µg/L, or ferritin 100–299 if TSAT < 20%)	IIa (weight of evidence is in favour of usefulness)/level A
American Heart Association/American College of Cardiology [47]	2016	In HF patients with NYHA class II and III and ID (ferritin < 100 ng/mL, or 100–300 ng/mL if TSAT < 20%), IV iron may be reasonable to improve functional status and quality of life	IIb (weak strength of recommendation)/ level B-R
Scottish Intercollegiate Guidelines Network [46]	2017	HFrEF patients with either NYHA class III and LVEF ≤ 45%; or NYHA class II and LVEF ≤ 40%, along with haemoglobin 9.5–13.5 g/dL should be considered for IV iron	1+ + + (high-quality meta-analyses, systematic reviews of RCTs, or RCTs with a very low risk of bias)
National Institute for Health and Care Excellence [48]	2018	No recommendation	

*HF* heart failure, *HFrEF* heart failure with reduced ejection fraction, *IV* intravenous, *ID* iron deficiency, *TSAT* transferrin saturation, *NYHA* New York Heart Association, *LVEF* left ventricular ejection fraction, *RCT* randomised controlled trial

## Conclusions

In conclusion, iron deficiency in heart failure is common and represents an independent predictor of poorer outcomes. IV iron does confer benefit in patients with HFrEF and concurrent ID, with several trials suggesting improvement with regards to hospitalisations for HF, NYHA functional class, quality of life scores, and exercise capacity (as measured by 6MWT distance and peak VO_2_). The evidence favouring iron replacement in acute decompensated HF is currently limited and less favourable, along with inadequate evidence for iron therapy in heart failure with preserved ejection fraction (HFpEF). IV iron appears to provide no benefit regarding CV mortality. Current guidelines recommend consideration of IV iron in those with concomitant HF and ID, and this is likely to expand once current ongoing trials provide further evidence regarding the potentially significant role of IV iron in patients with ID and HF.

Whilst IV iron therapy appears a promising addition to the arsenal of therapies for HF patients, before widespread adoption of IV iron in HF can occur several key questions must be answered. This includes the safety of routine, long-term use of IV iron in HFrEF; the efficacy of iron replacement therapy for HFpEF; whether long-term IV iron confers a benefit regarding mortality; and whether alternative iron preparations aside from FCM can provide any benefit. Moreover, many of these studies excluded patients with Hb values greater than 15 g//dL, hence the efficacy and safety of IV iron is unknown in these patients. There are currently four major ongoing trials which are attempting to answer many of the aforementioned questions, including FAIR-HFpEF exploring clinical outcomes for IV iron in HFpEF [50], IRONMAN exploring the use of iron (III) isomaltoside as an alternative to ferric carboxymaltose in HFrEF and ID [51], and FAIR-HF2 [52] and HEART-FID [53] exploring the long-term clinical effects of IV FCM in HFrEF. Table 3 contains more information regarding each trial. Positive results from these major studies would likely motivate stronger guideline recommendations regarding IV iron theray in routine practice.

**Table 3:** Major ongoing trials investigating intravenous iron in heart failure

	FAIR-HFpEF [50]	FAIR-HF2 [52]	IRONMAN [51]	HEART-FID [53]
ClinicalTrials.gov identifier	NCT03074591	NCT03036462	NCT02642562	NCT03037931
Actual Study Start Date	August 1, 2017	February 7, 2017	August 2016	March 15, 2017
Estimated study completion date	July 2021	December 2021	March 2022	March 2023
HF diagnosis	HFpEF	HFrEF	HFrEF	HFrEF
Number of participants	200	1200	1300	3014
Randomisation and preparation	1:1 FCM: placebo	1:1 FCM: placebo	1:1 Iron (III) isomaltoside: placebo	1:1 FCM: placebo
Blinding	Double-blind	Double-blind	Open label	Double-blind
Primary Outcomes	Change in 6-MWT distance from baseline to week 24	Combined rate of recurrent hospitalisations for HF and of CV death from baseline to at least 12 months	CV mortality or hospitalisation for worsening HF for a minimum of 6 months after last patient recruited	Incidence of death and incidence of hospitalisation for HF at 1 year Change in 6-MWT distance from baseline to 6 months
Duration	1 year	Event-driven; min. 1 year	Min. 2.5 years (average 3 years per participant)	Event driven; min 1 year
Important inclusion criteria	HFpEF with LVEF ≥ 45% NYHA class II or III Either hospitalised for HF within 12 months or raised NT-proBNP Hb > 9.0 g/dL and ≤ 14.0 g/dL	Chronic heart failure for at least 12 months Serum Hb 9.5–14 g/dL	LVEF < 45% NYHA class II–IV Current or recent (within 6 months) hospitalisation for HF Raised NT-proBNP	LVEF ≤ 40% NYHA II–IV Hb > 9.0 g/dL and < 13.5 g/dL (females) or < 15.0 g/dL (males)Either hospitalised for HF within 12 months or elevated NT-proBNP
Definition of ID	Serum ferritin < 100 ng/mL, or ferritin 100–299 plus TSAT < 20%	Serum ferritin < 100 ng/ml, or serum ferritin 100–299 ng/ml with TSAT < 20%	TSAT < 20% and/or ferritin < 100 ug/L	Ferritin < 100 ng/mL or 100 to 300 ng/mL with TSAT < 20%
Dosage	500–2000 mg FCM according to Hb and weight	1000–2000 mg FCM according to Hb and weight	Iron (III) isomaltoside 1,000-2000 mg according to Hb and body weight	Up to 750–1500 mg FCM according to Hb and weight

*HF* heart failure, *CV* cardiovascular, *HFrEF* heart failure with reduced ejection fraction, *HFpEF* heart failure with preserved ejection fraction, *FCM* ferrous carboxymaltose, *6-MWT* 6-min walk test, *TSAT* transferrin saturation, *NYHA* New York Heart Association, *LVEF* left ventricular ejection fraction, *NT = proBNP* N-terminal prohormone of brain natriuretic peptide, *Hb* haemoglobin, *min* minimum

IV iron was considered safe to use, with the above-mentioned studies all reporting similar side-effect profiles between treatment and control arms. No unacceptable side-effect or adverse-event profiles were observed with ferric carboxymaltose or iron sucrose. This concurs with a meta-analysis which reported IV iron was not associated with increased risk of serious AEs, and actually noted a reduction in serious AEs in HF patients (RR, 0.45; 95% CI, 0.29–0.70; I(2) = 0%) [54].

Lastly, the beneficial impact of IV iron in HFrEF was independent of baseline haemoglobin levels, hence patients with HF in the absence of anaemia should still have ferritin and TSAT measurements taken for consideration of iron therapy. The hypothesised reason behind this is because ID results in impaired mitochondrial adenosine triphosphate (ATP) synthesis long before it impacts haemopoiesis to levels detectable on a blood film. There is debate regarding whether the current definition of ID is optimal, and that using TSAT alone might give a better indication of true ID, because of ferritin’s role as an acute phase reactant [20, 49]. Additionally, a TSAT < 19.8% was shown to provide prognostic benefit and independently correlate with greater mortality [20]. This is significant because the administration of iron therapy to non-iron deficient patients is unlikely to provide benefit.

A major limitation of the studies discussed in this review was blinding. Because of IV iron’s characteristic dark-brown colour, the double-blinded studies would have been unable to achieve true blinding [24, 34–36, 39]. To mitigate this, black syringes were used along with curtains to prevent patients or healthcare professionals from deducing which infusion was connected. Using a solution of similar colour would have proven more reputable. Furthermore, many of the trials had small patient populations (< 500) and short follow-up durations. Whilst ongoing trials have not addressed the issue of blinding (all utilising normal saline, aside from IRONMAN which is open-label), three of the four have recruited > 1200 participans, and all have a mimumum follow-up duration of 1 year, with IRONMAN 2.5–3 years. Another limitation involves the lack of objective radiological evidence, such as echocardiography, surrounding the effects of iron replacement therapy on myocardium. Such a trial may reveal the extent of benefit (or lack thereof) of IV iron in heart failure.

## Data Availability

Data sharing is not applicable to this article as no datasets were generated or analysed during this literature review.

## Abbreviations

HF: Heart failure
ID: Iron deficiency
IV: Intravenous
HFrEF: Heart failure with reduced ejection fraction
NYHA: New York Heart Association
6MWT: 6-Minute walk test
HFpEF: Heart failure with preserved ejection fraction
Hb: Haemoglobin
RES: Reticuloendothelial system
TSAT: Transferrin saturation
NT-proBNP: N-terminal prohormone of brain natriuretic peptide
Tfr1: Transferrin receptor 1
QoL: Quality of life
FCM: Ferrous carboxymaltose
IS: Iron sucrose
PGA: Patient global assessment
AE: Adverse events
BNP: B-type natriuretic peptide
CV: Cardiovascular
ESC: European Society of Cardiology
SIGN: Scottish Intercollegiate Guidelines Network
ACC: American College of Cardiology
AHA: American Heart Association
ATP: Adenosine triphosphate
FERRIC-HF: Effect of intravenous iron sucrose on exercise tolerance in anemic and nonanemic patients with symptomatic chronic heart failure and iron deficiency
FAIR-HF: Ferinject assessment in patients with IRon deficiency and chronic heart failure trial
CONFIRM-HF: Ferric CarboxymaltOse evaluatioN on perFormance in patients with IRon deficiency in coMbination with chronic heart failure
EFFECT-HF: The effect of ferric carboxymaltose on exercise capacity in patients with chronic heart failure and iron deficiency
PRACTICE-ASIA-HF: Single-dose intravenous iron in Southeast Asian heart failure patients: a pilot randomized placebo-controlled study
AFFIRM-AHF: A randomised, double-blind placebo controlled trial comparing the effect of intravenous ferric carboxymaltose on hospitalisations and mortality in iron deficient patients admitted for acute heart failure
